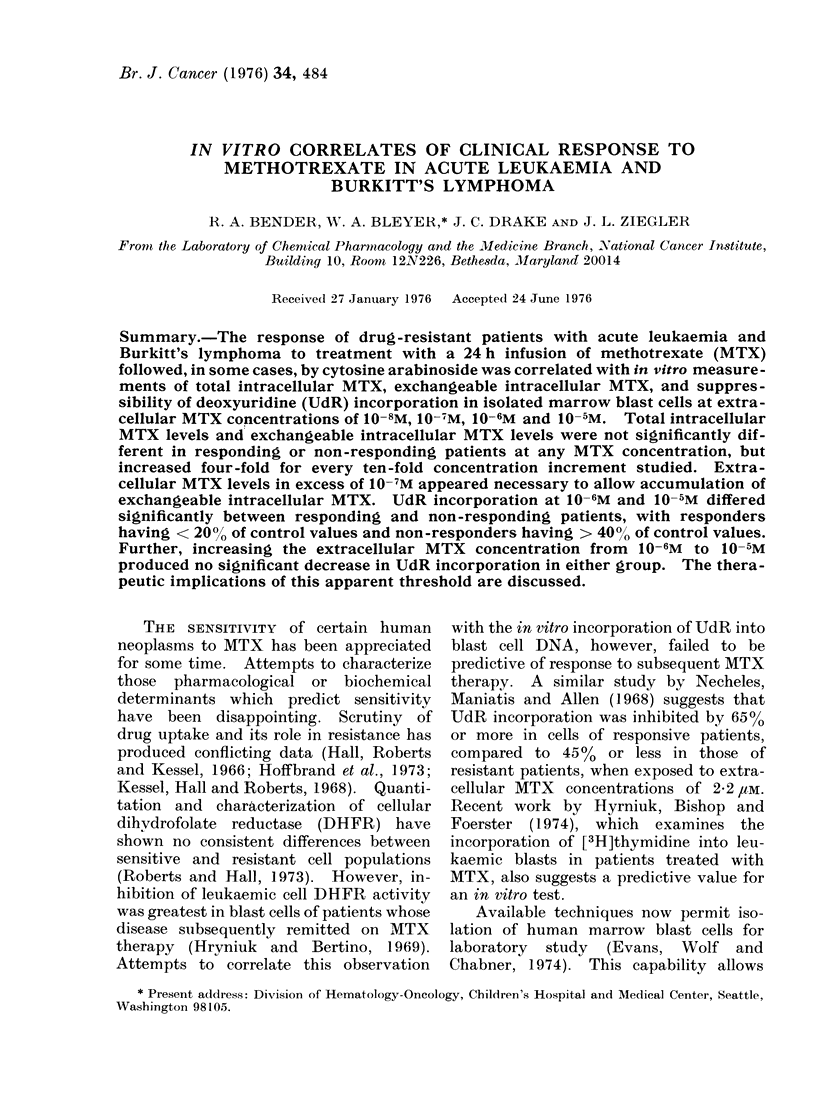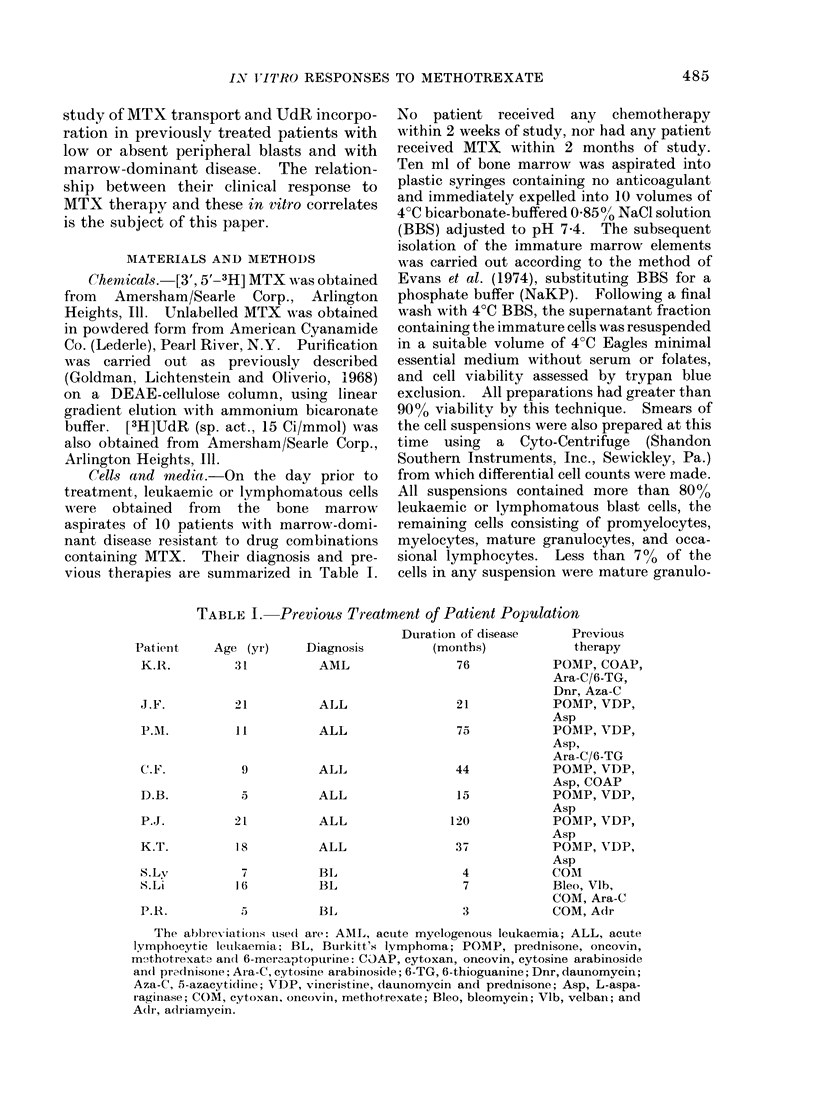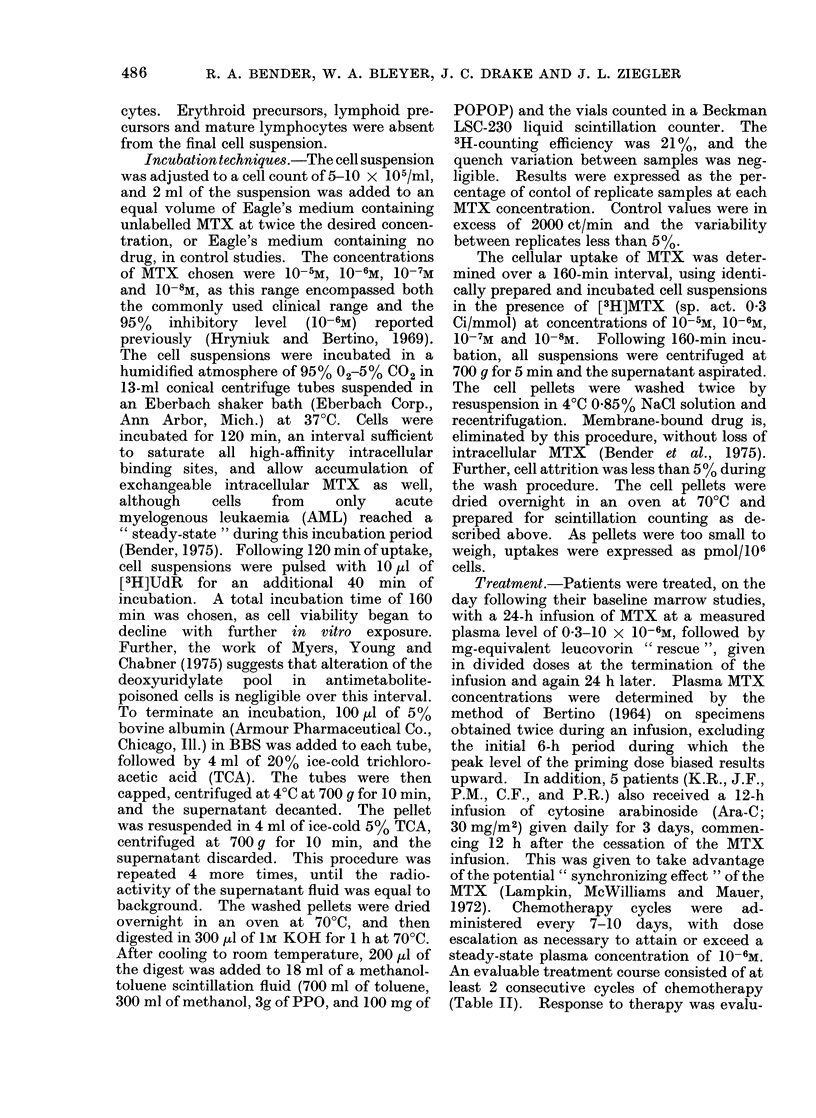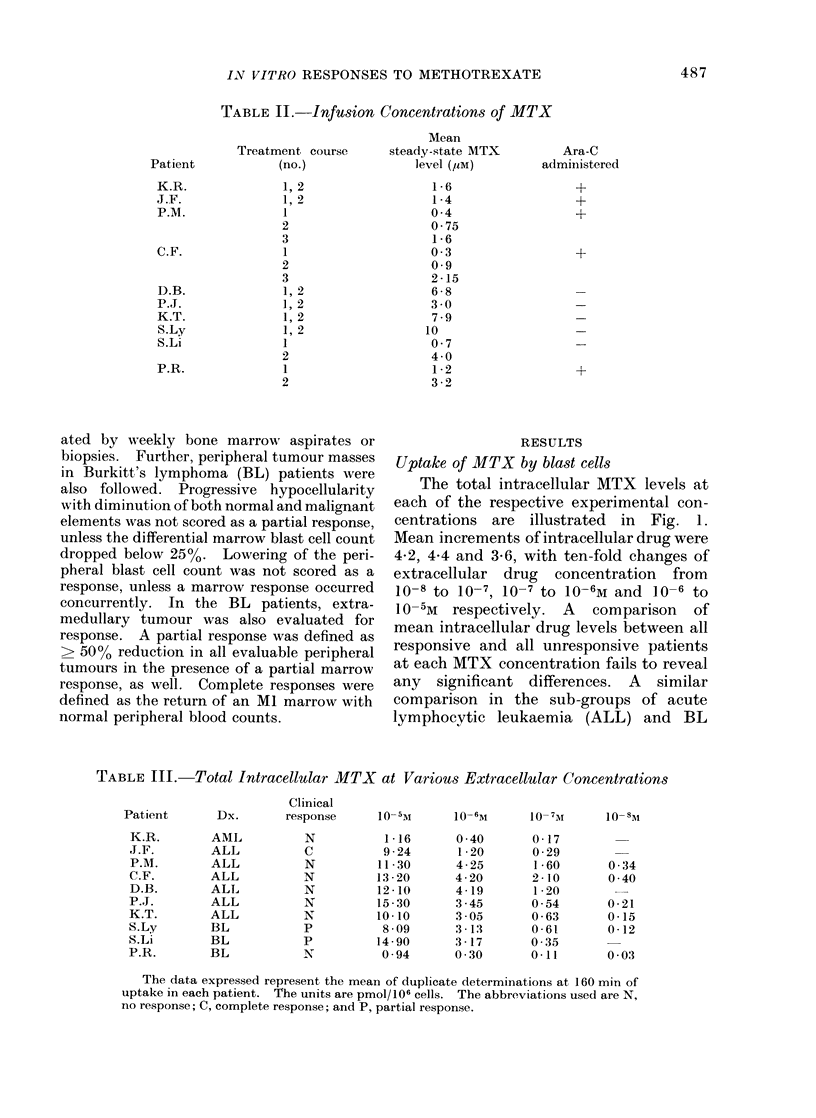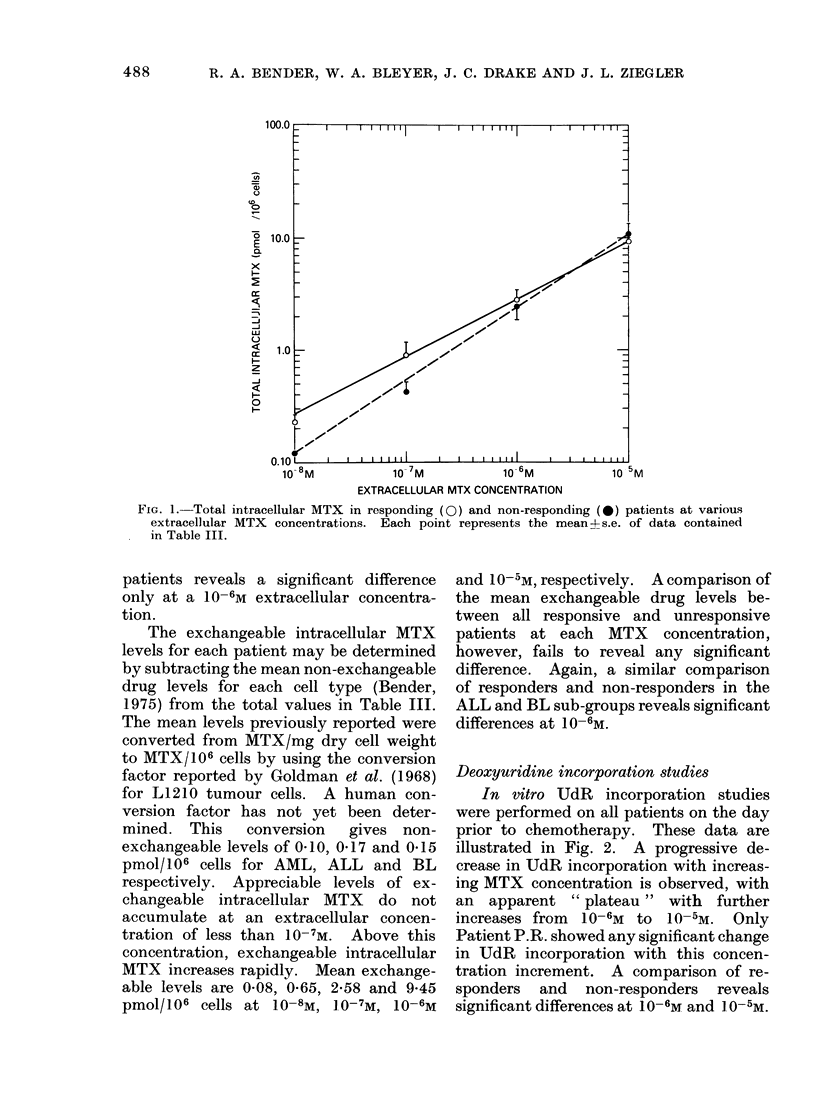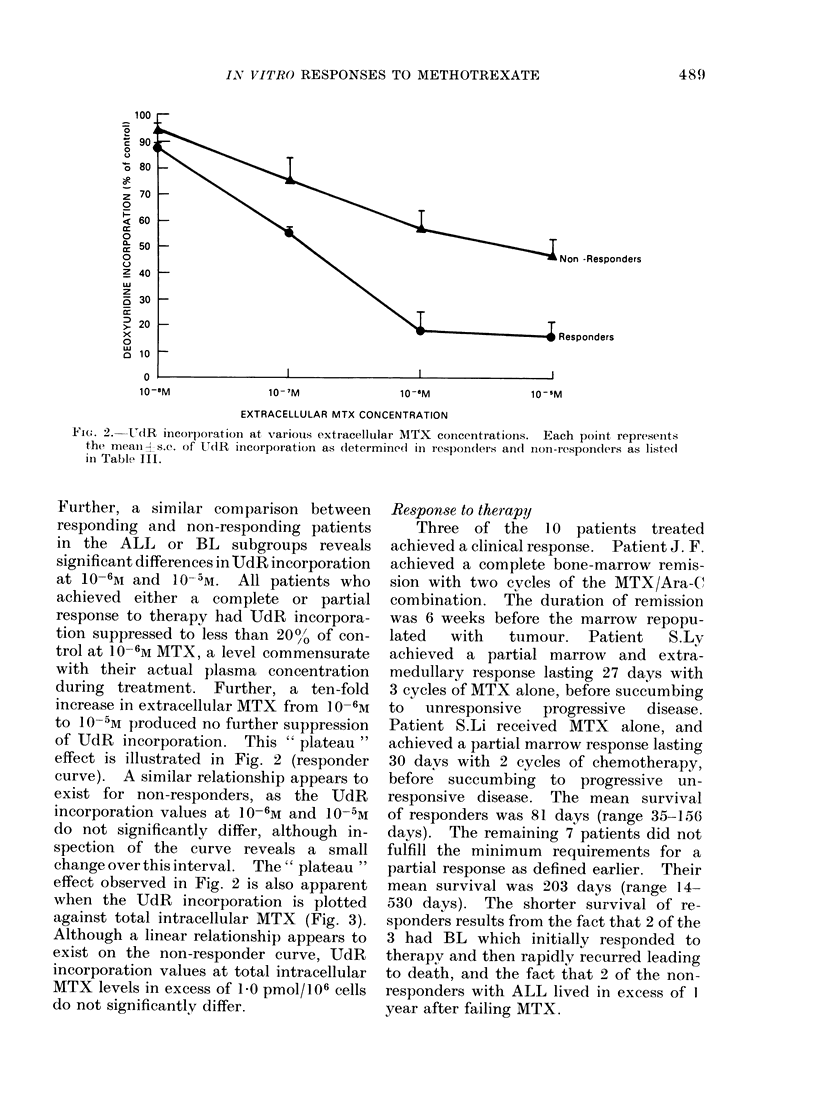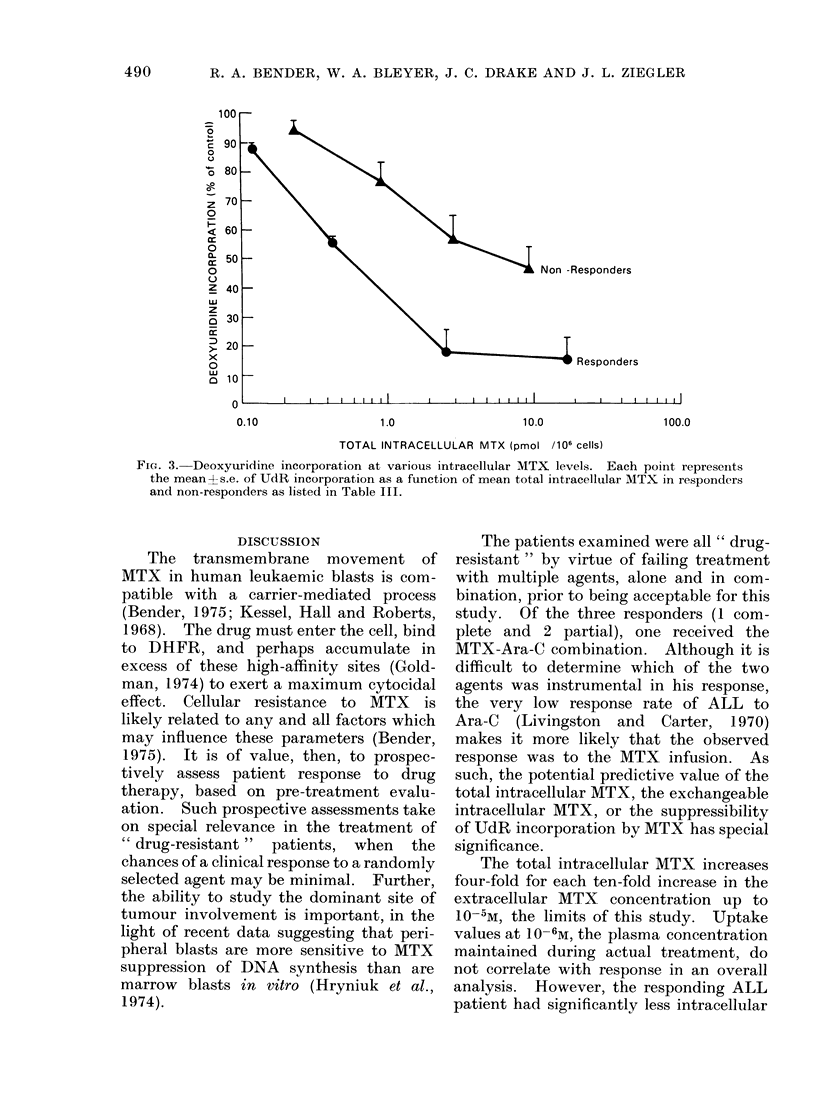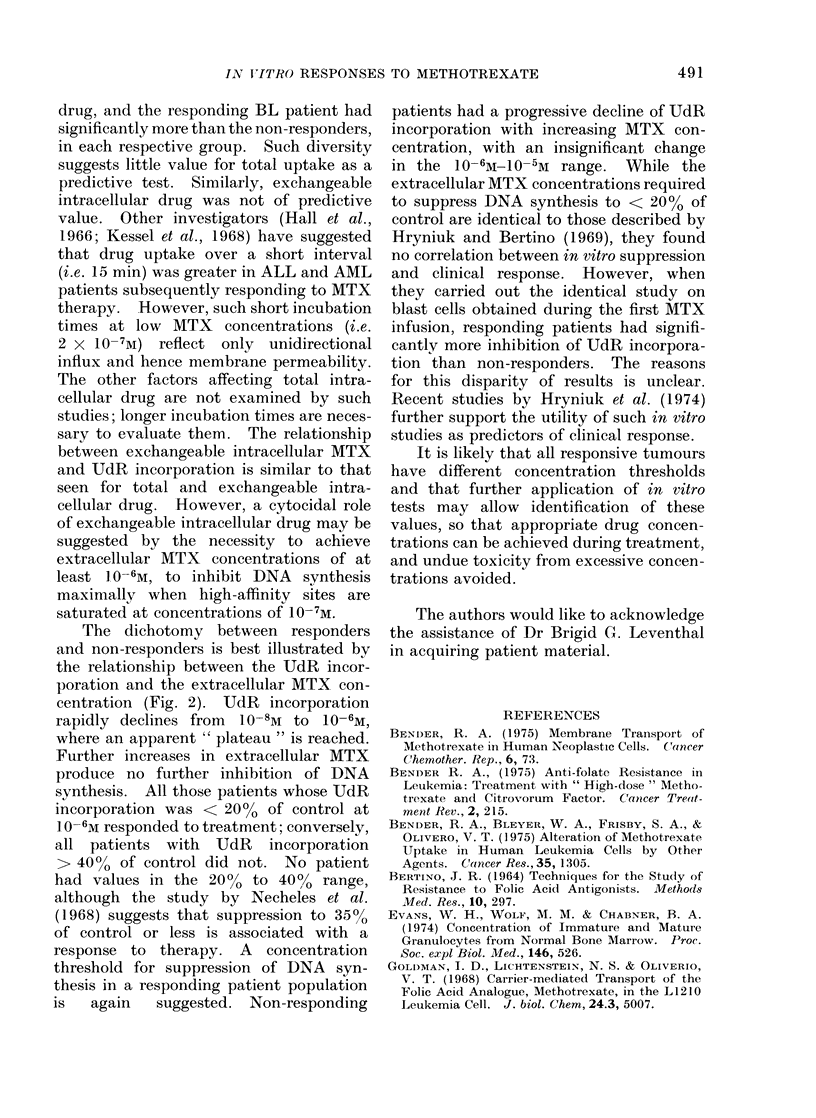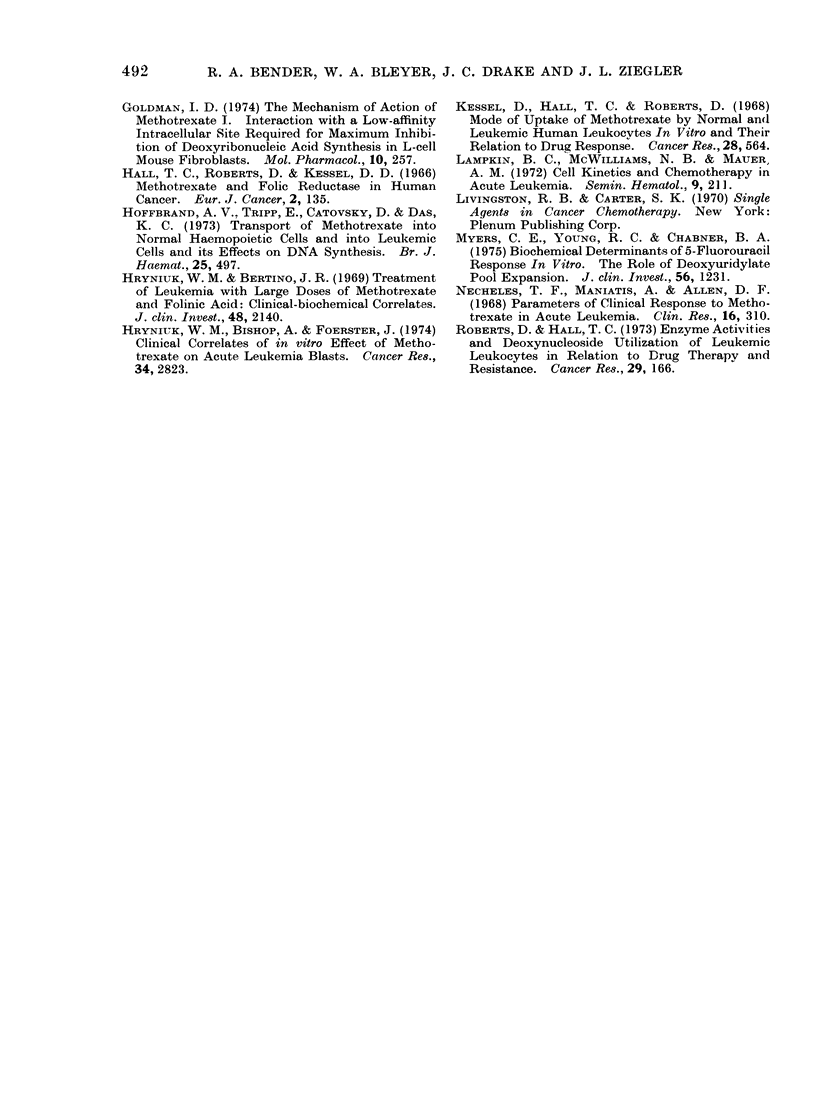# In vitro correlates of clinical response to methotrexate in actue leukaemia and Burkitt's lymphoma.

**DOI:** 10.1038/bjc.1976.202

**Published:** 1976-11

**Authors:** R. A. Bender, W. A. Bleyer, J. C. Drake, J. L. Ziegler

## Abstract

The response of drug-resistant patients with acute leukaemia and Burkitt's lymphoma to treatment with a 24 h infusion of methotrexate (MTX) followed, in some cases,by cytosine arabinoside was correlated with in vitro measurements of total intracellular MTX, exchangeable intracellular MTX, and suppressibility of deoxyuridine (UdR) incorporation in isolated marrow blast cells at extracellular MTX concentrations of 10(-8)M, 10(-7)M, 10(-6)M and 10(-5)M. Total intracellular MTX levels and exchangeable intracellular MTX levels were not significantly different in responding or non-responding patients at any MTX concentration, but increased four-fold for every ten-fold concentration increment studied. Extracellular MTX levels in excess of 10(-7)M appeared necessary to allow accumulation of exchangeable intracellular MTX. UdR incorporation at 10(-6)M and 10(-5)M differed significantly between responding and non-responding patients, with responders having less than 20% of control values and non-responders having greater than 40% of control values. Further, increasing the extracellular MTX concentration from 10(-6)M to 10(-5)M produced no significant decrease in UdR incorporation in either group. The therapeutic implications of this apparent threshold are discussed.


*Br. J. Cancer* (1976) **34**, 484

### *IN VITRO* CORRELATES OF CLINICAL RESPONSE TO METHOTREXATE IN ACUTE LEUKAEMIA AND BURKITT'S LYMPHOMA

R. A. BENDER, W. A. BLEYER,* J. C. DRAKE AND J. L. ZIEGLER

*From the Laboratory of Chemical Pharmacology and the Medicine Branch, National Cancer Institute, Building* 10, *Room* 12*N*226, *Bethesda, Maryland* 20014

Received 27 January 1976 Accepted 24 June 1976

**Summary.—The response of drug-resistant patients with acute leukaemia and Burkitt's lymphoma to treatment with a 24 h infusion of methotrexate (MTX) followed, in some cases, by cytosine arabinoside was correlated with *in vitro* measurements of total intracellular MTX, exchangeable intracellular MTX, and suppressibility of deoxyuridine (UdR) incorporation in isolated marrow blast cells at extracellular MTX concentrations of $10^{-8}$M, $10^{-7}$M, $10^{-6}$M and $10^{-5}$M. Total intracellular MTX levels and exchangeable intracellular MTX levels were not significantly different in responding or non-responding patients at any MTX concentration, but increased four-fold for every ten-fold concentration increment studied. Extracellular MTX levels in excess of $10^{-7}$M appeared necessary to allow accumulation of exchangeable intracellular MTX. UdR incorporation at $10^{-6}$M and $10^{-5}$M differed significantly between responding and non-responding patients, with responders having $< 20\%$ of control values and non-responders having $> 40\%$ of control values. Further, increasing the extracellular MTX concentration from $10^{-6}$M to $10^{-5}$M produced no significant decrease in UdR incorporation in either group. The therapeutic implications of this apparent threshold are discussed.**

THE SENSITIVITY of certain human neoplasms to MTX has been appreciated for some time. Attempts to characterize those pharmacological or biochemical determinants which predict sensitivity have been disappointing. Scrutiny of drug uptake and its role in resistance has produced conflicting data (Hall, Roberts and Kessel, 1966; Hoffbrand *et al.*, 1973; Kessel, Hall and Roberts, 1968). Quantitation and characterization of cellular dihydrofolate reductase (DHFR) have shown no consistent differences between sensitive and resistant cell populations (Roberts and Hall, 1973). However, inhibition of leukaemic cell DHFR activity was greatest in blast cells of patients whose disease subsequently remitted on MTX therapy (Hryniuk and Bertino, 1969). Attempts to correlate this observation with the *in vitro* incorporation of UdR into blast cell DNA, however, failed to be predictive of response to subsequent MTX therapy. A similar study by Necheles, Maniatis and Allen (1968) suggests that UdR incorporation was inhibited by 65% or more in cells of responsive patients, compared to 45% or less in those of resistant patients, when exposed to extracellular MTX concentrations of 2·2 $\mu$M. Recent work by Hyrniuk, Bishop and Foerster (1974), which examines the incorporation of [$^3$H]thymidine into leukaemic blasts in patients treated with MTX, also suggests a predictive value for an *in vitro* test.

Available techniques now permit isolation of human marrow blast cells for laboratory study (Evans, Wolf and Chabner, 1974). This capability allows

* Present address: Division of Hematology-Oncology, Children's Hospital and Medical Center, Seattle, Washington 98105.

study of MTX transport and UdR incorporation in previously treated patients with low or absent peripheral blasts and with marrow-dominant disease. The relationship between their clinical response to MTX therapy and these *in vitro* correlates is the subject of this paper.

### MATERIALS AND METHODS

*Chemicals.*—[3′, 5′-$^3$H] MTX was obtained from Amersham/Searle Corp., Arlington Heights, Ill. Unlabelled MTX was obtained in powdered form from American Cyanamide Co. (Lederle), Pearl River, N.Y. Purification was carried out as previously described (Goldman, Lichtenstein and Oliverio, 1968) on a DEAE-cellulose column, using linear gradient elution with ammonium bicaronate buffer. [$^3$H]UdR (sp. act., 15 Ci/mmol) was also obtained from Amersham/Searle Corp., Arlington Heights, Ill.

*Cells and media.*—On the day prior to treatment, leukaemic or lymphomatous cells were obtained from the bone marrow aspirates of 10 patients with marrow-dominant disease resistant to drug combinations containing MTX. Their diagnosis and previous therapies are summarized in Table I. No patient received any chemotherapy within 2 weeks of study, nor had any patient received MTX within 2 months of study. Ten ml of bone marrow was aspirated into plastic syringes containing no anticoagulant and immediately expelled into 10 volumes of 4°C bicarbonate-buffered 0·85% NaCl solution (BBS) adjusted to pH 7·4. The subsequent isolation of the immature marrow elements was carried out according to the method of Evans *et al.* (1974), substituting BBS for a phosphate buffer (NaKP). Following a final wash with 4°C BBS, the supernatant fraction containing the immature cells was resuspended in a suitable volume of 4°C Eagles minimal essential medium without serum or folates, and cell viability assessed by trypan blue exclusion. All preparations had greater than 90% viability by this technique. Smears of the cell suspensions were also prepared at this time using a Cyto-Centrifuge (Shandon Southern Instruments, Inc., Sewickley, Pa.) from which differential cell counts were made. All suspensions contained more than 80% leukaemic or lymphomatous blast cells, the remaining cells consisting of promyelocytes, myelocytes, mature granulocytes, and occasional lymphocytes. Less than 7% of the cells in any suspension were mature granulo-

TABLE I.—*Previous Treatment of Patient Population*

| Patient | Age (yr) | Diagnosis | Duration of disease (months) | Previous therapy |
|---|---|---|---|---|
| K.R. | 31 | AML | 76 | POMP, COAP, Ara-C/6-TG, Dnr, Aza-C |
| J.F. | 21 | ALL | 21 | POMP, VDP, Asp |
| P.M. | 11 | ALL | 75 | POMP, VDP, Asp, Ara-C/6-TG |
| C.F. | 9 | ALL | 44 | POMP, VDP, Asp, COAP |
| D.B. | 5 | ALL | 15 | POMP, VDP, Asp |
| P.J. | 21 | ALL | 120 | POMP, VDP, Asp |
| K.T. | 18 | ALL | 37 | POMP, VDP, Asp |
| S.Ly | 7 | BL | 4 | COM |
| S.Li | 16 | BL | 7 | Bleo, Vlb, COM, Ara-C |
| P.R. | 5 | BL | 3 | COM, Adr |

The abbreviations used are: AML, acute myelogenous leukaemia; ALL, acute lymphocytic leukaemia; BL, Burkitt's lymphoma; POMP, prednisone, oncovin, methotrexate and 6-mercaptopurine: COAP, cytoxan, oncovin, cytosine arabinoside and prednisone; Ara-C, cytosine arabinoside; 6-TG, 6-thioguanine; Dnr, daunomycin; Aza-C, 5-azacytidine; VDP, vincristine, daunomycin and prednisone; Asp, L-asparaginase; COM, cytoxan, oncovin, methotrexate; Bleo, bleomycin; Vlb, velban; and Adr, adriamycin.

cytes. Erythroid precursors, lymphoid precursors and mature lymphocytes were absent from the final cell suspension.

*Incubation techniques.*—The cell suspension was adjusted to a cell count of 5–10 × $10^5$/ml, and 2 ml of the suspension was added to an equal volume of Eagle's medium containing unlabelled MTX at twice the desired concentration, or Eagle's medium containing no drug, in control studies. The concentrations of MTX chosen were $10^{-5}$M, $10^{-6}$M, $10^{-7}$M and $10^{-8}$M, as this range encompassed both the commonly used clinical range and the 95% inhibitory level ($10^{-6}$M) reported previously (Hryniuk and Bertino, 1969). The cell suspensions were incubated in a humidified atmosphere of 95% $0_2$–5% $CO_2$ in 13-ml conical centrifuge tubes suspended in an Eberbach shaker bath (Eberbach Corp., Ann Arbor, Mich.) at 37°C. Cells were incubated for 120 min, an interval sufficient to saturate all high-affinity intracellular binding sites, and allow accumulation of exchangeable intracellular MTX as well, although cells from only acute myelogenous leukaemia (AML) reached a " steady-state " during this incubation period (Bender, 1975). Following 120 min of uptake, cell suspensions were pulsed with 10 µl of [$^3$H]UdR for an additional 40 min of incubation. A total incubation time of 160 min was chosen, as cell viability began to decline with further *in vitro* exposure. Further, the work of Myers, Young and Chabner (1975) suggests that alteration of the deoxyuridylate pool in antimetabolite-poisoned cells is negligible over this interval. To terminate an incubation, 100 µl of 5% bovine albumin (Armour Pharmaceutical Co., Chicago, Ill.) in BBS was added to each tube, followed by 4 ml of 20% ice-cold trichloroacetic acid (TCA). The tubes were then capped, centrifuged at 4°C at 700 *g* for 10 min, and the supernatant decanted. The pellet was resuspended in 4 ml of ice-cold 5% TCA, centrifuged at 700 *g* for 10 min, and the supernatant discarded. This procedure was repeated 4 more times, until the radioactivity of the supernatant fluid was equal to background. The washed pellets were dried overnight in an oven at 70°C, and then digested in 300 µl of 1M KOH for 1 h at 70°C. After cooling to room temperature, 200 µl of the digest was added to 18 ml of a methanol-toluene scintillation fluid (700 ml of toluene, 300 ml of methanol, 3g of PPO, and 100 mg of POPOP) and the vials counted in a Beckman LSC-230 liquid scintillation counter. The $^3$H-counting efficiency was 21%, and the quench variation between samples was negligible. Results were expressed as the percentage of contol of replicate samples at each MTX concentration. Control values were in excess of 2000 ct/min and the variability between replicates less than 5%.

The cellular uptake of MTX was determined over a 160-min interval, using identically prepared and incubated cell suspensions in the presence of [$^3$H]MTX (sp. act. 0·3 Ci/mmol) at concentrations of $10^{-5}$M, $10^{-6}$M, $10^{-7}$M and $10^{-8}$M. Following 160-min incubation, all suspensions were centrifuged at 700 *g* for 5 min and the supernatant aspirated. The cell pellets were washed twice by resuspension in 4°C 0·85% NaCl solution and recentrifugation. Membrane-bound drug is, eliminated by this procedure, without loss of intracellular MTX (Bender *et al.*, 1975). Further, cell attrition was less than 5% during the wash procedure. The cell pellets were dried overnight in an oven at 70°C and prepared for scintillation counting as described above. As pellets were too small to weigh, uptakes were expressed as pmol/$10^6$ cells.

*Treatment.*—Patients were treated, on the day following their baseline marrow studies, with a 24-h infusion of MTX at a measured plasma level of 0·3–10 × $10^{-6}$M, followed by mg-equivalent leucovorin " rescue ", given in divided doses at the termination of the infusion and again 24 h later. Plasma MTX concentrations were determined by the method of Bertino (1964) on specimens obtained twice during an infusion, excluding the initial 6-h period during which the peak level of the priming dose biased results upward. In addition, 5 patients (K.R., J.F., P.M., C.F., and P.R.) also received a 12-h infusion of cytosine arabinoside (Ara-C; 30 mg/m$^2$) given daily for 3 days, commencing 12 h after the cessation of the MTX infusion. This was given to take advantage of the potential " synchronizing effect " of the MTX (Lampkin, McWilliams and Mauer, 1972). Chemotherapy cycles were administered every 7–10 days, with dose escalation as necessary to attain or exceed a steady-state plasma concentration of $10^{-6}$M. An evaluable treatment course consisted of at least 2 consecutive cycles of chemotherapy (Table II). Response to therapy was evalu-

TABLE II.—*Infusion Concentrations of MTX*

| Patient | Treatment course (no.) | Mean steady-state MTX level (μM) | Ara-C administered |
|---|---|---|---|
| K.R. | 1, 2 | 1·6 | + |
| J.F. | 1, 2 | 1·4 | + |
| P.M. | 1 | 0·4 | + |
| | 2 | 0·75 | |
| | 3 | 1·6 | |
| C.F. | 1 | 0·3 | + |
| | 2 | 0·9 | |
| | 3 | 2·15 | |
| D.B. | 1, 2 | 6·8 | — |
| P.J. | 1, 2 | 3·0 | — |
| K.T. | 1, 2 | 7·9 | — |
| S.Ly | 1, 2 | 10 | — |
| S.Li | 1 | 0·7 | — |
| | 2 | 4·0 | |
| P.R. | 1 | 1·2 | + |
| | 2 | 3·2 | |

ated by weekly bone marrow aspirates or biopsies. Further, peripheral tumour masses in Burkitt's lymphoma (BL) patients were also followed. Progressive hypocellularity with diminution of both normal and malignant elements was not scored as a partial response, unless the differential marrow blast cell count dropped below 25%. Lowering of the peripheral blast cell count was not scored as a response, unless a marrow response occurred concurrently. In the BL patients, extramedullary tumour was also evaluated for response. A partial response was defined as $\geq$ 50% reduction in all evaluable peripheral tumours in the presence of a partial marrow response, as well. Complete responses were defined as the return of an M1 marrow with normal peripheral blood counts.

### RESULTS

### *Uptake of MTX by blast cells*

The total intracellular MTX levels at each of the respective experimental concentrations are illustrated in Fig. 1. Mean increments of intracellular drug were 4·2, 4·4 and 3·6, with ten-fold changes of extracellular drug concentration from $10^{-8}$ to $10^{-7}$, $10^{-7}$ to $10^{-6}$M and $10^{-6}$ to $10^{-5}$M respectively. A comparison of mean intracellular drug levels between all responsive and all unresponsive patients at each MTX concentration fails to reveal any significant differences. A similar comparison in the sub-groups of acute lymphocytic leukaemia (ALL) and BL

TABLE III.—*Total Intracellular MTX at Various Extracellular Concentrations*

| Patient | Dx. | Clinical response | $10^{-5}$M | $10^{-6}$M | $10^{-7}$M | $10^{-8}$M |
|---|---|---|---|---|---|---|
| K.R. | AML | N | 1·16 | 0·40 | 0·17 | — |
| J.F. | ALL | C | 9·24 | 1·20 | 0·29 | — |
| P.M. | ALL | N | 11·30 | 4·25 | 1·60 | 0·34 |
| C.F. | ALL | N | 13·20 | 4·20 | 2·10 | 0·40 |
| D.B. | ALL | N | 12·10 | 4·19 | 1·20 | — |
| P.J. | ALL | N | 15·30 | 3·45 | 0·54 | 0·21 |
| K.T. | ALL | N | 10·10 | 3·05 | 0·63 | 0·15 |
| S.Ly | BL | P | 8·09 | 3·13 | 0·61 | 0·12 |
| S.Li | BL | P | 14·90 | 3·17 | 0·35 | — |
| P.R. | BL | N | 0·94 | 0·30 | 0·11 | 0·03 |

The data expressed represent the mean of duplicate determinations at 160 min of uptake in each patient. The units are pmol/$10^6$ cells. The abbreviations used are N, no response; C, complete response; and P, partial response.

FIG. 1.—Total intracellular MTX in responding (○) and non-responding (●) patients at various extracellular MTX concentrations. Each point represents the mean ± s.e. of data contained in Table III.

patients reveals a significant difference only at a $10^{-6}$M extracellular concentration.

The exchangeable intracellular MTX levels for each patient may be determined by subtracting the mean non-exchangeable drug levels for each cell type (Bender, 1975) from the total values in Table III. The mean levels previously reported were converted from MTX/mg dry cell weight to MTX/$10^6$ cells by using the conversion factor reported by Goldman *et al.* (1968) for L1210 tumour cells. A human conversion factor has not yet been determined. This conversion gives non-exchangeable levels of 0·10, 0·17 and 0·15 pmol/$10^6$ cells for AML, ALL and BL respectively. Appreciable levels of exchangeable intracellular MTX do not accumulate at an extracellular concentration of less than $10^{-7}$M. Above this concentration, exchangeable intracellular MTX increases rapidly. Mean exchangeable levels are 0·08, 0·65, 2·58 and 9·45 pmol/$10^6$ cells at $10^{-8}$M, $10^{-7}$M, $10^{-6}$M and $10^{-5}$M, respectively. A comparison of the mean exchangeable drug levels between all responsive and unresponsive patients at each MTX concentration, however, fails to reveal any significant difference. Again, a similar comparison of responders and non-responders in the ALL and BL sub-groups reveals significant differences at $10^{-6}$M.

### *Deoxyuridine incorporation studies*

*In vitro* UdR incorporation studies were performed on all patients on the day prior to chemotherapy. These data are illustrated in Fig. 2. A progressive decrease in UdR incorporation with increasing MTX concentration is observed, with an apparent " plateau " with further increases from $10^{-6}$M to $10^{-5}$M. Only Patient P.R. showed any significant change in UdR incorporation with this concentration increment. A comparison of responders and non-responders reveals significant differences at $10^{-6}$M and $10^{-5}$M.

FIG. 2.—UdR incorporation at various extracellular MTX concentrations. Each point represents the mean ± s.e. of UdR incorporation as determined in responders and non-responders as listed in Table III.

Further, a similar comparison between responding and non-responding patients in the ALL or BL subgroups reveals significant differences in UdR incorporation at $10^{-6}$M and $10^{-5}$M. All patients who achieved either a complete or partial response to therapy had UdR incorporation suppressed to less than 20% of control at $10^{-6}$M MTX, a level commensurate with their actual plasma concentration during treatment. Further, a ten-fold increase in extracellular MTX from $10^{-6}$M to $10^{-5}$M produced no further suppression of UdR incorporation. This " plateau " effect is illustrated in Fig. 2 (responder curve). A similar relationship appears to exist for non-responders, as the UdR incorporation values at $10^{-6}$M and $10^{-5}$M do not significantly differ, although inspection of the curve reveals a small change over this interval. The " plateau " effect observed in Fig. 2 is also apparent when the UdR incorporation is plotted against total intracellular MTX (Fig. 3). Although a linear relationship appears to exist on the non-responder curve, UdR incorporation values at total intracellular MTX levels in excess of 1·0 pmol/$10^6$ cells do not significantly differ.

*Response to therapy*

Three of the 10 patients treated achieved a clinical response. Patient J. F. achieved a complete bone-marrow remission with two cycles of the MTX/Ara-C combination. The duration of remission was 6 weeks before the marrow repopulated with tumour. Patient S.Ly achieved a partial marrow and extramedullary response lasting 27 days with 3 cycles of MTX alone, before succumbing to unresponsive progressive disease. Patient S.Li received MTX alone, and achieved a partial marrow response lasting 30 days with 2 cycles of chemotherapy, before succumbing to progressive unresponsive disease. The mean survival of responders was 81 days (range 35–156 days). The remaining 7 patients did not fulfill the minimum requirements for a partial response as defined earlier. Their mean survival was 203 days (range 14–530 days). The shorter survival of responders results from the fact that 2 of the 3 had BL which initially responded to therapy and then rapidly recurred leading to death, and the fact that 2 of the non-responders with ALL lived in excess of 1 year after failing MTX.

FIG. 3.—Deoxyuridine incorporation at various intracellular MTX levels. Each point represents the mean ± s.e. of UdR incorporation as a function of mean total intracellular MTX in responders and non-responders as listed in Table III.

### DISCUSSION

The transmembrane movement of MTX in human leukaemic blasts is compatible with a carrier-mediated process (Bender, 1975; Kessel, Hall and Roberts, 1968). The drug must enter the cell, bind to DHFR, and perhaps accumulate in excess of these high-affinity sites (Goldman, 1974) to exert a maximum cytocidal effect. Cellular resistance to MTX is likely related to any and all factors which may influence these parameters (Bender, 1975). It is of value, then, to prospectively assess patient response to drug therapy, based on pre-treatment evaluation. Such prospective assessments take on special relevance in the treatment of " drug-resistant " patients, when the chances of a clinical response to a randomly selected agent may be minimal. Further, the ability to study the dominant site of tumour involvement is important, in the light of recent data suggesting that peripheral blasts are more sensitive to MTX suppression of DNA synthesis than are marrow blasts *in vitro* (Hryniuk *et al.*, 1974).

The patients examined were all " drug-resistant " by virtue of failing treatment with multiple agents, alone and in combination, prior to being acceptable for this study. Of the three responders (1 complete and 2 partial), one received the MTX-Ara-C combination. Although it is difficult to determine which of the two agents was instrumental in his response, the very low response rate of ALL to Ara-C (Livingston and Carter, 1970) makes it more likely that the observed response was to the MTX infusion. As such, the potential predictive value of the total intracellular MTX, the exchangeable intracellular MTX, or the suppressibility of UdR incorporation by MTX has special significance.

The total intracellular MTX increases four-fold for each ten-fold increase in the extracellular MTX concentration up to $10^{-5}$M, the limits of this study. Uptake values at $10^{-6}$M, the plasma concentration maintained during actual treatment, do not correlate with response in an overall analysis. However, the responding ALL patient had significantly less intracellular drug, and the responding BL patient had significantly more than the non-responders, in each respective group. Such diversity suggests little value for total uptake as a predictive test. Similarly, exchangeable intracellular drug was not of predictive value. Other investigators (Hall *et al.*, 1966; Kessel *et al.*, 1968) have suggested that drug uptake over a short interval (*i.e.* 15 min) was greater in ALL and AML patients subsequently responding to MTX therapy. However, such short incubation times at low MTX concentrations (*i.e.* $2 \times 10^{-7}$M) reflect only unidirectional influx and hence membrane permeability. The other factors affecting total intracellular drug are not examined by such studies; longer incubation times are necessary to evaluate them. The relationship between exchangeable intracellular MTX and UdR incorporation is similar to that seen for total and exchangeable intracellular drug. However, a cytocidal role of exchangeable intracellular drug may be suggested by the necessity to achieve extracellular MTX concentrations of at least $10^{-6}$M, to inhibit DNA synthesis maximally when high-affinity sites are saturated at concentrations of $10^{-7}$M.

The dichotomy between responders and non-responders is best illustrated by the relationship between the UdR incorporation and the extracellular MTX concentration (Fig. 2). UdR incorporation rapidly declines from $10^{-8}$M to $10^{-6}$M, where an apparent " plateau " is reached. Further increases in extracellular MTX produce no further inhibition of DNA synthesis. All those patients whose UdR incorporation was $< 20\%$ of control at $10^{-6}$M responded to treatment; conversely, all patients with UdR incorporation $> 40\%$ of control did not. No patient had values in the 20% to 40% range, although the study by Necheles *et al.* (1968) suggests that suppression to 35% of control or less is associated with a response to therapy. A concentration threshold for suppression of DNA synthesis in a responding patient population is again suggested. Non-responding patients had a progressive decline of UdR incorporation with increasing MTX concentration, with an insignificant change in the $10^{-6}$M–$10^{-5}$M range. While the extracellular MTX concentrations required to suppress DNA synthesis to $< 20\%$ of control are identical to those described by Hryniuk and Bertino (1969), they found no correlation between *in vitro* suppression and clinical response. However, when they carried out the identical study on blast cells obtained during the first MTX infusion, responding patients had significantly more inhibition of UdR incorporation than non-responders. The reasons for this disparity of results is unclear. Recent studies by Hryniuk *et al.* (1974) further support the utility of such *in vitro* studies as predictors of clinical response.

It is likely that all responsive tumours have different concentration thresholds and that further application of *in vitro* tests may allow identification of these values, so that appropriate drug concentrations can be achieved during treatment, and undue toxicity from excessive concentrations avoided.

The authors would like to acknowledge the assistance of Dr Brigid G. Leventhal in acquiring patient material.

### REFERENCES

Bender, R. A. (1975) Membrane Transport of Methotrexate in Human Neoplastic Cells. *Cancer Chemother. Rep.*, **6**, 73.

Bender R. A., (1975) Anti-folate Resistance in Leukemia: Treatment with " High-dose " Methotrexate and Citrovorum Factor. *Cancer Treatment Rev.*, **2**, 215.

Bender, R. A., Bleyer, W. A., Frisby, S. A., & Olivero, V. T. (1975) Alteration of Methotrexate Uptake in Human Leukemia Cells by Other Agents. *Cancer Res.*, **35**, 1305.

Bertino, J. R. (1964) Techniques for the Study of Resistance to Folic Acid Antigonists. *Methods Med. Res.*, **10**, 297.

Evans, W. H., Wolf, M. M. & Chabner, B. A. (1974) Concentration of Immature and Mature Granulocytes from Normal Bone Marrow. *Proc. Soc. expl Biol. Med.*, **146**, 526.

Goldman, I. D., Lichtenstein, N. S. & Oliverio, V. T. (1968) Carrier-mediated Transport of the Folic Acid Analogue, Methotrexate, in the L1210 Leukemia Cell. *J. biol. Chem*, **24.3**, 5007.

GOLDMAN, I. D. (1974) The Mechanism of Action of Methotrexate I. Interaction with a Low-affinity Intracellular Site Required for Maximum Inhibition of Deoxyribonucleic Acid Synthesis in L-cell Mouse Fibroblasts. *Mol. Pharmacol.*, **10**, 257.

HALL, T. C., ROBERTS, D. & KESSEL, D. D. (1966) Methotrexate and Folic Reductase in Human Cancer. *Eur. J. Cancer*, **2**, 135.

HOFFBRAND, A. V., TRIPP, E., CATOVSKY, D. & DAS, K. C. (1973) Transport of Methotrexate into Normal Haemopoietic Cells and into Leukemic Cells and its Effects on DNA Synthesis. *Br. J. Haemat.*, **25**, 497.

HRYNIUK, W. M. & BERTINO, J. R. (1969) Treatment of Leukemia with Large Doses of Methotrexate and Folinic Acid: Clinical-biochemical Correlates. *J. clin. Invest.*, **48**, 2140.

HRYNIUK, W. M., BISHOP, A. & FOERSTER, J. (1974) Clinical Correlates of *in vitro* Effect of Methotrexate on Acute Leukemia Blasts. *Cancer Res.*, **34**, 2823.

KESSEL, D., HALL, T. C. & ROBERTS, D. (1968) Mode of Uptake of Methotrexate by Normal and Leukemic Human Leukocytes *In Vitro* and Their Relation to Drug Response. *Cancer Res.*, **28**, 564.

LAMPKIN, B. C., MCWILLIAMS, N. B. & MAUER, A. M. (1972) Cell Kinetics and Chemotherapy in Acute Leukemia. *Semin. Hematol.*, **9**, 211.

LIVINGSTON, R. B. & CARTER, S. K. (1970) *Single Agents in Cancer Chemotherapy*. New York: Plenum Publishing Corp.

MYERS, C. E., YOUNG, R. C. & CHABNER, B. A. (1975) Biochemical Determinants of 5-Fluorouracil Response *In Vitro*. The Role of Deoxyuridylate Pool Expansion. *J. clin. Invest.*, **56**, 1231.

NECHELES, T. F., MANIATIS, A. & ALLEN, D. F. (1968) Parameters of Clinical Response to Methotrexate in Acute Leukemia. *Clin. Res.*, **16**, 310.

ROBERTS, D. & HALL, T. C. (1973) Enzyme Activities and Deoxynucleoside Utilization of Leukemic Leukocytes in Relation to Drug Therapy and Resistance. *Cancer Res.*, **29**, 166.